# Interactions with regulatory antibodies modulate CHIP E3 ubiquitin ligase‐driven tau clearance

**DOI:** 10.1002/alz.086539

**Published:** 2025-01-03

**Authors:** Aparna Unnikrishnan, Emily J Connelly, Dong hee Chung, Cory M Nadel, Aye Thwin, Charles S Craik, Daniel Southworth

**Affiliations:** ^1^ University of California San Francisco, San Francisco, CA USA

## Abstract

**Background:**

The direct and chaperone‐associated interactions of E3 ubiquitin ligase CHIP with tau in Alzheimer’s disease and other tauopathies, regulates tau turnover, by directly linking it to ubiquitination and proteasomal degradation, as well as through suppression of tau aggregation. Modulation of these CHIP‐driven tau clearance mechanisms can be an effective treatment strategy. Antigen‐binding antibody fragments (Fabs) are potent tools that can highly‐selectively engage target proteins and act as functional probes or inhibitors. Development and characterization of Fabs that target unique 3D epitopes on CHIP can help in exploring their therapeutic potential, while enabling structural characterization and providing insights into CHIP‐driven tau clearance mechanisms.

**Method:**

Recombinant Fabs were developed to conformation‐selectively bind CHIP, by bio‐panning and BLI binding assays. They were then functionally screened using SEC‐MALS, ubiquitination activity assay, binding assays, ThT fibrillation assays etc. Selected Fab interactions that potently regulated CHIP‐driven mechanisms were further characterized via Cryo‐EM high‐resolution structural studies.

**Result:**

CHIP‐Fab complexes with distinct 3D binding epitopes and different functional regulatory effects were identified and studied. High‐resolution details of these Fab interaction sites on CHIP were obtained by Cryo‐EM 3D reconstruction and molecular modeling. Fab ‘2F1’ that forms a 2:2 complex with CHIP, potently inhibits its ubiquitination activity by directly blocking the required E2‐CHIP interactions. Fab ‘H1’ that forms both 1:2 and 2:2 complexes with CHIP, significantly improves its tau aggregation suppression and reveals distinct functional conformational states of CHIP. Fab ‘2D2’ forms a 1:2 complex with CHIP, blocks CHIP‐tau interactions and stabilizes the CHIP coiled‐coil domain dimerization interface.

**Conclusion:**

Functional screening of Fabs that were generated to specifically target 3D epitopes on CHIP have revealed potent modes of CHIP functional modulation. Insights from the corresponding CHIP‐Fab complex structures help in better understanding the CHIP‐driven tau clearance mechanisms and in describing unique therapeutic target sites on CHIP.

